# Kurzfristig aufgetreten Läsionen an der Fußsohle – ist etwas Unheilvolles im Gange?

**DOI:** 10.1007/s00105-026-05701-6

**Published:** 2026-05-21

**Authors:** Yasmin Fathi, Rainer Hofmann-Wellenhof

**Affiliations:** Univ. Klinik für Dermatologie und Venerologie, Medizinische Universität Graz, Auenbruggerplatz 8, 8010 Graz, Österreich

Ein 44-jahriger Mann stellte sich wegen zweier schwarzer Läsionen auf der rechten Fußsohle vor. Diese bestünden erst seit 2 Wochen, Druckschmerzen hätte er aber schon seit einem Jahr an dieser Stelle verspürt.

Bei der Untersuchung zeigte sich auf der Sohle des rechten Fußes eine 3 × 1,5 cm großes asymmetrisches, livide Makula mit 2 kleineren, asymmetrisch gelegenen schwarzen Papeln mit einem Durchmesser von etwa 4  bzw. 7 mm (Abb. 1). Dermatoskopisch war eine livide homogene Makula mit einem sehr diskreten hellbraunen Randsaum zu sehen. In dieser Makula waren 2 schwarze homogene Papeln sichtbar. Die größere Papel zeigte einige kleine Ulzerationen (Abb. 2)

**Abb. 1:**
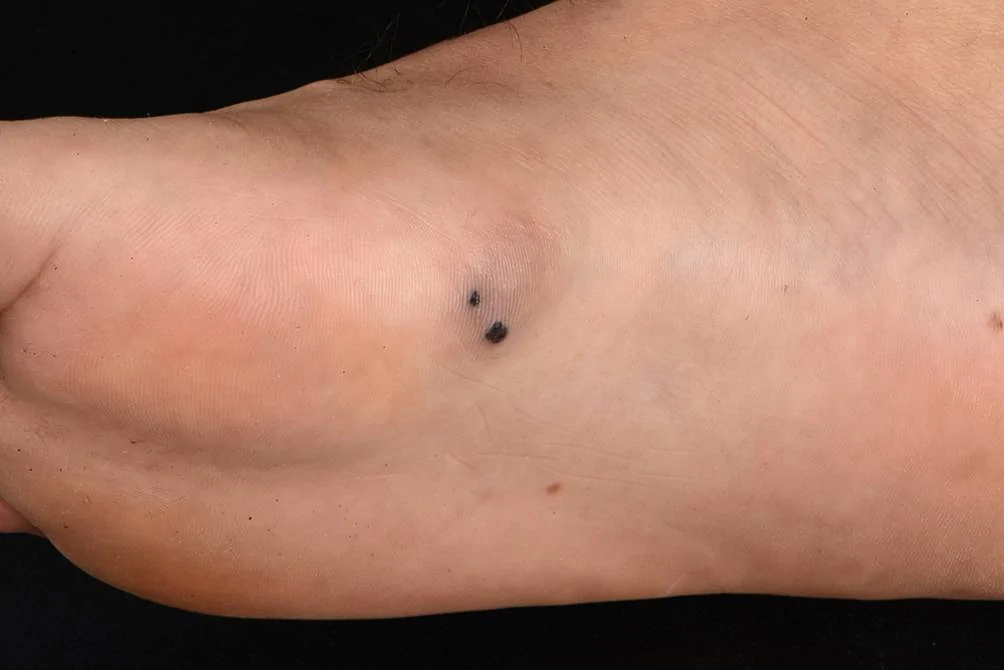
Livide Makula mit 2 schwarzen Papeln an der rechten Fußsohle

**Abb. 2:**
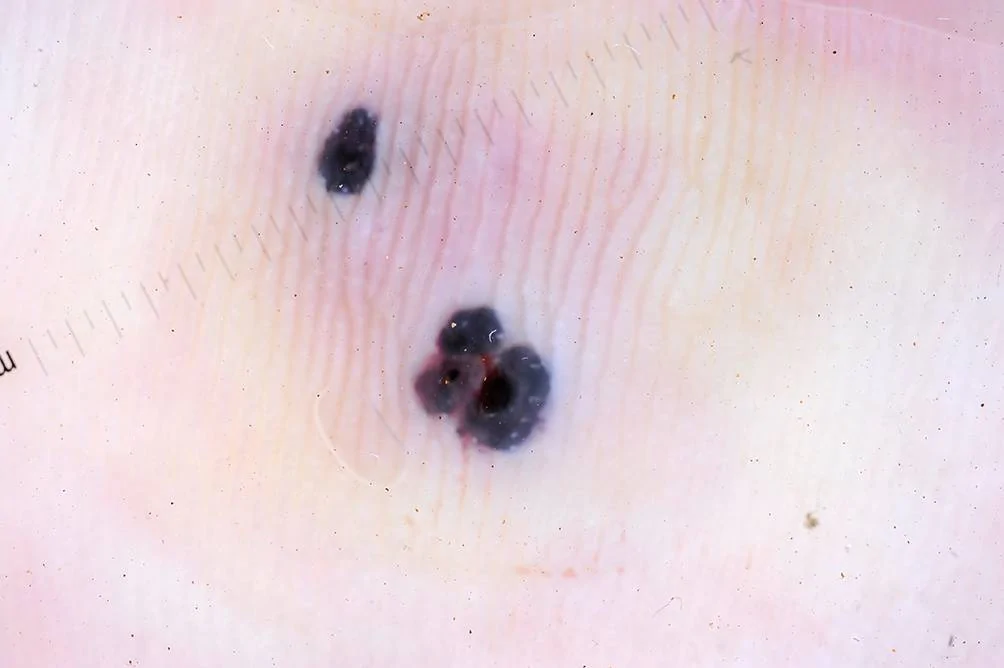
Dermatoskopie: livide Makula mit hellbraunem Rand. Innerhalb der Makula finden sich 2 schwarze Areale, das untere zeigt Ulzerationen

## Wie lautet Ihre Diagnose?

Die histologische Untersuchung ergab ein Angiokeratom.

## Diskussion

Angiokeratom ist ein Oberbegriff, der sich auf eine Gruppe von Kapillarfehlbildungen bezieht. Diese gutartigen Tumoren sind durch kapillare Ektasien der papillären Dermis gekennzeichnet, die zu Hyperkeratose, Papillomatosis und Akanthose der Epidermis führen. Es gibt viele unterschiedliche klinische Manifestationen [1].

Typischerweise treten Angiokeratome bei der Geburt als eine kleine rote Makula auf, die im Laufe der Zeit dunkel und warzenartig wird. Sie können überall am Körper auftreten, werden aber am häufigsten einseitig an den unteren Extremitäten als kleine Läsion von bis zu 5 mm gefunden. Die meisten Angiokeratome sind asymptomatisch, gelegentlich kommt es zu Blutungen, Juckreiz und Schmerzen [2].

Es gibt verschiedene Differenzialdiagnosen einer pigmentierten Läsion an der Fußsohle, wie das akrale Melanom oder das Angiosarkom.

Das akrale lentiginöse Melanom (ALM) ist ein Untertyp des Melanoms an Handflächen und Fußsohlen. Bei der Dermatoskopie zeigen ALMs typischerweise variable Pigmentierung, asymmetrische Muster und ein paralleles Leistenmuster [3]. Das Angiosarkom ist ein weiterer aggressiver bösartiger Tumor, der pigmentierte Läsionen bildet. In den frühen Stadien können Läsionen wie unklar definierte Blutergüsse erscheinen, die sich schnell zu ulzerierenden Knötchen entwickeln [4]. Das Kaposi-Sarkom tritt am häufigsten bei immungeschwächten Personen auf. Bei der Dermatoskopie kann das seltene, jedoch typische Regenbogenzeichen, eine spezielle Farbbrechung durch das polarisierende Licht, richtungsweisen für die Diagnose sein [5, 6].

Der typische Befund eines Angiokeratoms in der Dermatoskopie besteht in vaskulären Lakunen, die sich als Konfluenz von dunkelrotem Pigment zeigen. Diese war jedoch bei unserem Patienten nicht vorhanden, es zeigten sich nur schwarze Areale, die in der Histologie mit thrombosierten Gefäßen korrespondierten. Auch die Lokalisation an der Fußsohle ist für ein Angiokeratom ungewöhnlich und machte eine Biopsie notwendig.
